# The current role of surgery and SBRT in early stage of small cell lung cancer

**Published:** 2021-02-17

**Authors:** Núria Farré, José Belda-Sanchis, Mauro Guarino, Laura Tilea, Jady Vivian Rojas Cordero, Elisabeth Martínez-Téllez

**Affiliations:** ^1^Department of Radiation Oncology, Hospital de la Santa Creu i Sant Pau, Universitat Autònoma de Barcelona, Barcelona, Spain; ^2^Department of Thoracic Surgery, Hospital de la Santa Creu i Sant Pau, Universitat Autònoma de Barcelona, Barcelona, Spain,

**Keywords:** chemoradiotherapy, chemotherapy, early stage small cell lung cancer, prophylactic cranial irradiation, radiotherapy, small cell lung cancer, stereotactic ablative radiotherapy, stereotactic body radiotherapy, surgery

## Abstract

**Background:**

Early stage small cell lung cancer (T1-2N0M0SCLC) represents 7% of all SCLC. The standard treatment in patients with intrathoracic SCLC disease is the use of concurrent chemoradiotherapy (CRT). Nowadays, the recommended management of this highly selected group is surgical resection due to favorable survival outcomes. For medically inoperable patients or those who refuse surgery, there is an increasing interest in evaluating the role of Stereotactic Body Radiotherapy (SBRT) for T1-2N0SCLC, transferring the favorable experience obtained on inoperable NSCLC (Non-Small-cell Lung Cancer). In the era of multimodality treatment, adjuvant systemic therapy plays an important role even in the management of early SCLC, increasing the disease-free survival (DFS) and Overall Survival (OS). The benefit of Prophylactic Cranial Irradiation (PCI), that currently has a Category I recommendation for localized stage SLCL, remains controversial in this selected subgroup of patients due to the lower risk of brain metastasis.

**Aim:**

This review summarizes the most relevant data on the local management of T1-2N0M0SCLC (surgery and radiotherapy), and evaluates the relevance of adjuvant treatment.

**Relevance for patients:**

Provides a critical evaluation of best current clinical management options for T1-2N0M0 SCLC.

## 1. Introduction

Small cell lung cancer (SCLC) accounts for 15-20% of all lung cancers [1-3]. It is strongly associated with cigarette smoking and the most aggressive subtype with a median overall survival time of 7 months and 5-year relative survival rates of 5-7% [4,5]. SCLC is most often diagnosed at advanced stages. Using the Veterans Administration Lung Cancer Study Group stage system, roughly 60% of the patients present with limited-stage disease while 40% present with extensive stage [1]. Early-stage SCLC (T1-2N0SCLC), defined as Stages I and II according to the American Joint Committee on Cancer Staging [6,7], comprises 7% of all SCLC and 0.29% of all lung cancers [8].

Even for this selected subgroup of patients, the 5-year overall survival (OS) remains poor, <35%, despite an intensive treatment. At this moment, the international guidelines recommend a local therapy as the first option for early SCLC. Surgical resection for fit patients has shown favorable survival outcomes in several studies [9,10], whereas definitive radiotherapy (RT) treatment is recommended for inoperable patients in addition to systemic therapy. Recently, the development of a highly conformal RT treatment, Stereotactic Body Radiotherapy (SBRT) with excellent results in early NSCLC, has led to an increased interest in its use in early well-staged SCLC patients [11,12].

The authors present a literature review of the current role of surgery and SBRT in early stage small cell lung cancer to provide a critical evaluation of the available data from existing studies. A comprehensive search was conducted in the NHS evidence (www.evidence.nhs.uk), PubMed (www.ncbi.nlm.nih.gov/pmc/) and Embase (www.embase.com), from 1966 to 2019. Studies published in languages other than English were excluded. Search terms included: Early small cell lung cancer, Small cell lung cancer, Stereotactic body radiotherapy, Stereotactic ablative radiotherapy, and Surgery. Prospective, retrospective, meta-analysis, case–control, and case reports with more than 5 patients were included in the study.

## 2. Treatment of ES-SCLC. Surgery versus SBRT

### 2.1. Defining limited/early stage SCLC

In 1957, the Veterans Administration Lung Cancer Study Group (VALSG) established a dichotomous stage system for SCLC: Limited disease when the tumor was confined to one hemithorax and extended disease in case of bilateral pulmonary or extrathoracic involvement. From a practical point of view, a limited disease would be encompassed within an acceptable RT field, while the RT field required for treating the extended disease would be too large and would be associated with inadmissible toxicity. This simplified two-stage system, applied in clinical practice and trials during many years, has proven to be adequate for most clinical decisions and carries additional prognostic information [13]. In 1989, the IASLC proposed a modification to the VALSG classification of the limited disease including contralateral mediastinal or supraclavicular lymph node metastases and ipsilateral pleural effusions independent of cytology.

In 1993, Shepherd *et al*. points out the relevance of an accurate clinical staging of limited SCLC as a helpful system to differentiate prognostic subgroups and proposed the term of “very limited disease” for the SCLC with a negative cervical mediastinoscopy and/or no evidence of enlarged mediastinal lymph nodes on radiologic examinations [14].

The prognostic value of the TNM staging system has been observed in small series of patients undergoing surgical resection for SCLC, mainly in the setting of a multimodal therapy [14-22]. Nonetheless, the TNM staging system for SCLC was not formally recommended by the AJCC till 2009. In 2009 (7^th^ edition) and 2015 (8^th^ edition), based on the prognostic value of both clinical and pathological TNM staging, the International Union for Cancer Control (UICC) Classification of Malignant Tumors recommended the use of the TNM staging system for SCLC [23,24]. A recent study conducted by Abdel-Rahman validates both the prognostic value of TNM lung cancer staging system and the improvement over the prognostic value of the old veterans’ administration system [25].

Despite the above-mentioned, a considerable variation exists concerning which definition should be used for classifying SCLC, even in the context of multidisciplinary team meetings.

### 2.2. Surgery for ES-SCLC

#### 2.2.1. Surgery alone for ES-SCLC

Concurrent CRT is considered to be the standard of care for patients with ES-SCLC, resulting in a median survival of 17 months and an overall 5-year survival of 10% [1,26]. Historically, surgery alone has been considered to have a limited role in the primary treatment of early-stage SCLC as it had been associated with an overall 5-year survival close to 0%. In 1969, the British Medical Research Council Trial randomly allocated 144 operable patients with a SCLC diagnosed on bronchial biopsy to either surgical resection or RT. The mean and 10-year overall survival results showed that radical RT was associated to a moderately better result than surgery (300 days for radical RT vs. 199 days for surgery – *P*=0.04 – and 1% for radical RT vs. 0% for surgery). The conclusion of this study was that radical RT is preferable to surgery in the treatment of patients with SCLC [27-29]. These results were broadly in line with those published by Mountain *et al*. in 1974 [30] and Martini *et al*. in 1975 [31].

#### 2.2.2. Surgery within multimodality treatment for T1-2N0M0 SCLC

2.2.2.1. Rationale for surgery in early stage SCLC

Systemic therapy plus concurrent RT is considered the gold standard for patients with T1-2N0SCLC and RT is a crucial component for the local treatment. However, the local in-field failure rate after CRT is high and ranges from 30 to 70% [17,32-35]. In addition, despite having achieved complete radiologic response, up to 70% of these patients will have detectable residual disease in the resected specimen [32,36].

The above-mentioned highlights a possible role of surgery in the local treatment of T1-2N0SCLC. Many authors have addressed this matter [37-40]. The rationale for surgical resection in SCLC can be summarized as follows:

1. Intraoperative diagnosis on frozen section of SCLC of a solitary nodule not suspected to be a SCLC. Although this scenario is very unlikely due to the aggressiveness of the SCLC, there is a small subgroup of patients (3-5%) who presents initially with disease strictly limited to the lung [8]. In this subset of patients, aggressive treatment (anatomic resection, systematic lymphadenectomy, and adjuvant CT or CRT) can achieve similar survival rates to that of non–small-cell lung cancer.
  SCLC tend to be accurately diagnosed intraoperatively on frozen section, in some cases it can represent a difficult diagnostic problem [41]. In a series of 125 unexpected SCLC diagnosed after pulmonary resection, only 78 cases were diagnosed as SCLC by intraoperative frozen section examinations. The other 47 cases were diagnosed intraoperatively as poorly differentiated, non-specific lung cancer or carcinoid tumors. There was also inconsistencies between the clinical and pathological stage of these unexpected SCLC mainly caused by nodal upstaging, highlighting the importance of an adequate systematic nodal dissection [42].
2. Surgical resection for very limited-SCLC (defined as T1N0, T2N0) may improve local control compared to CRT [8-10,16,18,21,34,43-88]. The first sites of recurrence after complete remission are either the primary tumor region or the hilar and mediastinal lymph nodes. It has been demonstrated that local control after multimodality treatment including complete surgical resection is 95-100% [15,16,18,48,74,89,90].
  Accurate hilar and mediastinal staging are mandatory in this subgroup of patients with a potentially resectable SCLC. Ideally, the mediastinal staging should include invasive techniques as endobronchial ultrasonographic needle aspiration, endoscopic ultrasonographic needle aspiration, conventional and extended cervical mediastinoscopy, anterior mediastinotomy, and video-assisted thoracic surgery. Thomas *et al*. studied 477 patients with clinical Stage I SCLC (cT1-2aN0M0) from the National Cancer Database treated with curative-intent resection, followed by adjuvant therapy. Initial clinical and final pathological stages were compared to determine the upstaging rate; it was 25%, 30% due to a higher pathologic T descriptor, and 81% due to the presence of nodal disease. Overall 5-year survival was significantly worse for upstaged patients compared with those who remained a pathologically Stage I (36% vs. 52%, *P*<0.001) [60].
3. Combined histology tumors (a SCLC tumor with a NSCLC component). The incidence of combined SCLC ranges from 2% to 28% [91-95]. It is thought that relapse or failure to respond to CT may be due to the NSCLC component.
4. Salvage surgery for chemoresistant localized SCLC or local relapse after an initial response to C/CRT. In this scenario, selected patients may benefit from surgical resection more than a second-line CT [17,96,97].

2.2.2.2. Does surgical resection of T1-2N0SCLC improve overall survival?

The role of surgery in small cell lung cancer (SCLC) is controversial. Two old randomized controlled trials have shown no significant benefit from surgery compared to conventional treatment [36,98]. In the largest study conducted by Lad *et al.*, a total of 146 patients with SCLC received induction CT. Patients achieving at least partial response after induction and fit enough for thoracotomy were randomized to surgery or no intervention. Complete resection rate was 77%. All patients had chest and brain irradiation postoperatively. There was no difference in survival between the two groups. The conclusion of the authors was that surgery does not add any benefit in terms of survival or local disease control to multimodality treatment of SCLC. Nevertheless, quality of the evidence from these randomized trials is very low [99] and the results should be taken with caution as the staging pre-operative methods; pre-operative neoadjuvant therapy and surgical procedures used in these studies do not reflect current knowledge and clinical practice.

Ever since, many small series (largely retrospective, but also Phase II trials, meta-analysis, systematic reviews, and propensity matched score analysis) have shown excellent survival for patients with T1-2N0SCLC treated surgically, mainly in the setting of a multimodal approach [8-10,16,18,21,34,43-88].

Table 1 summarizes surgical and survival data for patients with SCLC published in the past 25 years.

**Table 1:** Surgical and survival dates for patients with SCLC (period time revised 1995-2020)

Study	Study type and time period LOE	Inclusion criteria	Number patients	Neoadjuvant/adjuvant treatments	PCI	Survival (Sv) data
Jin, 2018 [131]	RS, SEER 2004-2013 3A	cI-II	N=2129 S 387, RT 1032 S+RT 154 No S nor RT 556		-	5-year OS T1N0 46.0% versus 23.8% S versus RT 5-year OS T2N0: 42.6% versus 24.7% S versus RT T3N0 or T1-2N1 (stage IIB) SCLC patients who underwent S did not have higher 5-year OS and LCSS rates than patients who received RT
Ahmed, 2017 [46]	RS, SEER 2007-2013 3A	Stage I SCLC	N=1902 S 427, S+RT 115	-	-	MST 50 mos. (S) MST 60+mos. (S+RT)
Wakeam, 2017 [134]	RS, NCDB 2004-13 3A	cT1-2N0M0	N=5079		MST 25.3 mos.
Wakeam 2017 [53]	RS, NCDB 2004-2013 Stage-specific Propensity score match S versus NST 3A	cI-III	N=2619	No adjuvant treatment 24% NC or NR 4% AC 27%; AR 1% ACR 32%; NC or NR and AC or AR 2% Other 10%	_	MST Stage I 38.6 versus 22.9 mos. S versus NST MST Stage II 23.4 versus 20.7 mos. S versus NST MST stage IIIA21.7 versus 16.0 mos. S versus NST,
Combs, 2015 [102]	RS, NCDB1998-2011 3A	cT1-3N0-2 SCLC	N=2,476 S 841 cIA, 168 cIB	All S: 68%	-	5-year OS: 54% (cIA); 36% (cIB
Ogawa, 2012 [45]	RS, 1995-2008 Institutional 4	cI-III pI-III SCLC	N=28 (23 SCLC before S) S 21cI,5 cII,7 cIII2	NC 8 AC 19, ACR 2	-	5-year OS 47%
Ju, 2012 [79]	RS,1990-2009 Institutional 4	pI-III	N=34	NC 3 AC 1, AR 19, 10 CRT	-	5-year OS 66%
Vallières, 2009 [136]	RS, IASLC 1990-2000 3A	Resected SCLC	N=349 (68 pIA, 91 pIB)	-	-	5-year OS: 53% (pIA); 44% (pIB)
Wang, 2007 [47]	RS, Institutional 4	pI-III	N=122	CT and CRT (not specified)	-	MST 50 mos. 5-year OS 66%
Veronesi, 2007 [44]	RS, Institutional 4	cI-IIIA	N=23	AC all	-	MST 24 mos.
Tsuchiya, 2005 [83]	Prospective Phase II trial 1991-1996 2B	cI-IIIA	N=62	AC 42 (69%)	-	MST not reached in pI stage MST 449 days in pII stage MST 712 days in pIIIA disease. 3-year OS 61% 3-year Sv rate cI, cII, cIIIA stage 68%, 56%, and 13%, respectively
Brock, 2005 [72]	RS, Institutional 1976-2002 4	Resected SCLC	N=82 (24 Stage I→S + AC)	AC 55%	23%	5-year OS: 86% (platinum); 42% (non-platinum)
Nakamura, 2004 [43]	RS, Institutional 4	cI-III SCLC	N=69	S 37, NC 32, AC 41, ACR 7	-	5-year Sv 48.9% cI 33.3% cII 20.2% cIIIA 0% cIIIB.
Badzio, 2004 [70]	Comparative RS, Institutional 1984-1996 4	cI-III balanced in both, S and non-S groups	N=134	S 67 (all AC) Non-S 67 (all CT)	34% only S group	MST 22 mos. (S) MST 11 mos. (non-S) 5-year OS S 27%, non-S 4%
Lewinski, 2001 [20]	RS, Institutional 1976-2002 4	cI-IIIA SCLC	N=75 46 underwent thoracotomy and 35 lung resection	NC all	If CR to NC	MST N0+1 25 mos. MST N2 14 mos. MST resected 18 mos. 5-year OS resected 29%
Cataldo, 2000 [137]	RS, Institutional 1982-1992 4	cI-III SCLC	N=60	AC 88% pII AR (11%) pIII AR (21%)	41%	5-year Sv rate 40% pI, 36% pII, and 15% pIII.
Inoue, 2000 [18]	RS, Institutional 1975-1994 4	Resected SCLC	N=91 (32 cIA, 30 cIB)	All 78%	5.5%	MST 53 mos. 5-year OS 49% (cIA) MST 25 mos. 5-year OS 47% (cIB)
Kobayashi, 2000 [138]	RS, Institutional 1982-19922 4	cI-III SCLC	N=59	NC 71%	-	5-year survival rate 55% pI, 33% pII, 23% pIII.
Eberhardt, 1999 [48]	Prospective phase II trial. Institutional 1991-1995 2B	cIB-cIIIB	N=46	IB/IIA had NC+S IIB/IIIA had NCR+S	-	MST all patients 36 mos. MST R0 patients 68 mos. 5-year Sv rate all patients 46% 5-year Sv rate R0 patients 63%
Rea, 1998 [21]	RS, Institutional 1981-1995 4	cI -III SCLC	N=104	51 cI-II received S+ACR 53 cIII received NC+S + AR	35%	MST 28 mos. 5-year OS rate 32% 5-year OS pI 52.2%, pII 30% pIII 15.3%
Lucchi, 1997 [69]	RS, Institutional 1975-1995 4	Resected SCLC	N=1272001	15 S 92 S+AC+AR if N1-2 (34 patients) 15 NC	10%	MST 18 mos. 5-year actuarial Sv rate 22.6%
Fujimori, 1997 [49]	RS, Institutional 1987-1993 4	cI-IIIA SCLC	N=22	NC all	–	MST 62 mos. 3-year OS rate67% 3-y OS ratecI-II 73% 3-year OS rate cIII 43%
Wada, 1995 [139]	RS, Institutional 1976-1991 4	cI -III SCLC	N=46	NC+AC 37.0% AC 50.5% the S 12,5%	_	5-year Sv rate cI-II who received NC 80.0% 5-year Sv rate cI-II who received AC 37.7% 5-year Sv rate cIII-IIIb who received NC 10.0% 5-year Sv rate cIIIa or IIIb who received AC 0.0%
Karrer and Ulsperger, 1995 [75]	Prospective RCT ISC-LCSG 2B	T12N0M0 SCLC	N=183	AC	100%	2.5-year Sv rate 63% for 68 patients after R0-pTN0M0 2.5-year Sv rate 37% for 27 patients after R0-pTN2M0

SCLC: Small cell lung cancer; PCI: Prophylactic cranial irradiation; RS: Retrospective study; SEER: Surveillance, epidemiology, and end results database; ISC-LCSG: The Lung Cancer Study Group of the International Society of Chemotherapy; S: Surgery; CT: Chemotherapy; RT: Radiotherapy; NCDB, National Cancer Data Base; cIA, clinical stage IA; cIB, clinical stage IB IASLC: International Association for the study of Lung Cancer; pI (A): Pathologic stage IA; pI (B), pathologic stage I (B); pII: pathologic stage II; pIII (A): Pathologic stage IIIA; pIII (B): pathologic stage IIIB; R0: Complete resection; AC: adjuvant chemotherapy; MST, median survival time; NC: neoadjuvant chemotherapy; ACR: adjuvant chemoradiotherapy. CR: complete response; NST: non-surgical treatment; A dash represents lack of information or details; LOE, Level of evidence (From the Centre for Evidence-Based Medicine, http://www.cebm.net.)

Ahmed *et al*. identified 1902 patients with Stage I SCLC from the SEER database. Of these, 28.5% underwent surgical resection, a percentage that remained unchanged from 2007 to 2013 despite improved outcomes with surgical resection as part of a multimodal approach. The authors point out the significant disparities in the management of patients with Stage I SCLC and the need to educate physicians on the importance to consider surgical resection for this subgroup of patients [46].

Jones *et al*. conducted a best evidence topic published in 2013 concluding that surgery would be indicated in clinical Stages I or II SCLC after an accurate pre-operative staging of mediastinal lymph nodes including a mediastinoscopy. In this scenario, surgery for T1-2N0SCLC improves the prognosis as part of a multimodal treatment. However, patients with Stage III disease are unlikely to benefit from surgery [100,101].

The effectiveness of surgery in patients with locally advanced SCLC (Stages II and III) has been specifically addressed in both retrospective series of cases [10,18,48,74,87,89,100,102] and propensity score matching analyses [103]. Data from these studies show that surgery is highly effective in local control and is associated with a significant overall survival benefit after complete resection in the context of a multimodal treatment including neoadjuvant/adjuvant chemoradiation and prophylactic cranial irradiation (PCI). Nevertheless, the favorable survival associated to the surgery-based multi-modality treatment appears to be stage-dependent, ranging from 20 to 57% (5-year overall probability of survival), with a detrimental impact on hazard ratio for OS over the stages.

With the aim of assessing the potential role of surgery for pathologically confirmed SCLC, Staged T1-4 N0-2 M0 (AJCC 8^th^ edition), Wei *et al*. used propensity score-matching analysis to compare overall survival (OS) in a matched cohort of 1562 patients from the SEER database (2004-2014). In the matched cohort, surgery was associated with a significant 5-year OS improvement (from 16.8% to 36.7%, *P*<0.001). After adjustment for confounders using a Cox regression model, survival benefit of surgery was significant in all subgroups, including N1-2 disease, except for patients with a tumor size > 5.0 cm or T3 disease. The authors concluded that selected patients (T size <5 cm/T1-2) with SCLC would benefit from surgery, including N1-2 disease [104].

Du *et al*. conducted another propensity score matching analysis of patients with SCLC from the SEER database (2010-2015) to investigate the impact of surgery on survival. A total of 1,707 patients were included in the matched cohort. The authors found that patients who did not receive surgery had an increased risk of death when compared with patients who did. The results support that patients with SCLC with Stage I-IIA (T1-2N0M0) and selected IIB (N1) may benefit from surgery [105]. Similar results were found by Peng *et al*. in regard to the impact of surgery on lung cancer specific survival in a cohort of 2453 patients with early stage SCLC from the SEER database [106]. Yu *et al*. analyzed a series of 1560 patients with stage I SCLC from the SEER database, of which 399 treated surgically (242 underwent lobectomy, 121 had local tumor excision/ablation, ten had a pneumonectomy, and unknown type of surgery in 21). Most of the surgical group of patients did not receive radiation therapy. The authors found that surgery, even without RT, seems to offer acceptable OS outcomes for Stage I patients who undergo lobectomy (5-year OS 50.3%, 95% CI 43.1-57.1%, for the group of lobectomies without RT and 57.1%, 95% CI 37.4-72.7%, for the group of lobectomies with RT) [65].

Wakeam *et al*. performed a propensity score matching analysis of patients with SCLC from the National Cancer Database. A total of 2089 patients with a SCLC clinically Staged I-IIIA were matched. The study aimed to evaluate outcomes of curative surgery in comparison to CT-based non-surgical treatment. Surgery was associated with longer survival across all the stages. In a sub-analyses by T and N descriptors, significant differences in OS was observed in favor of patients who underwent surgical resection ofT3/T4 N0 tumors (median OS 33.0 vs. 16.8 months, *P*=0.008), N1 (24.4 vs. 18.3 months *P*=0.03), and N2 tumors (20.1 vs. 14.6 months, *P*=0.007). The authors conclude that surgical resection is associated with significantly longer survival for early SCLC [53].

Regarding the type of resection, there is evidence suggesting that patients who underwent a lobectomy had significant better OS over sublobar resection [8,53,65,67,86,87,90,102,105]. In the retrospective study of SCLC patients in the National Cancer Data Base published by Combs *et al.*, lobectomy was associated with a 5-year overall survival of 40% compared with 21% for sublobar and 22% for pneumonectomy (HR for death after sublobar resections vs. lobectomy 1.38,95% CI 1.12-1.71) [102]. Similar results were found in the propensity-matched analysis of survival of patients from the same database published by Wakeam *et al*. [53] comparing resection of Stage I-IIIA SCLC with CT-based non-surgical treatment, as well as in the systematic review and meta-analysis of the role of surgery in Stage I to III SCLC published by Liu *et al*. [66] and in the study of Schreiber *et al*. [87] using the SEER database to analyze survival outcomes of patients who underwent surgery with both localized disease and regional SCLC. Du *et al*. conducted a propensity score matching analysis of patients with SCLC included in the SEER database from 2010 to 2015 and concluded as well that patients who received a sublobar resection had a significant increased risk of mortality when compared with patients who received a lobectomy (*P*=0.03) [105].

2.2.2.3. What do guidelines say on surgical treatment of T1-2N0SCLC?

There is not a complete agreement on the indication of surgery in T1-2N0 SCLC between the different guidelines. The National Comprehensive Cancer Network (NCCN) [107], ASTRO [108], and ASCO [109] guidelines recommend surgery as the initial treatment option in operable patients having a Stage T1-2N0M0 (I-IIA) SCLC. The ESMO Clinical Practice Guidelines (108) recommend surgical treatment for patients with a SCLC stage T1-2N0-1M0. All the guidelines state that indication of surgical treatment and RT should be based on the American Joint Committee on Cancer/Union for International Cancer Control TNM Staging System.

An accurate pre-operative mediastinal staging is universally recommended before indicating surgical treatment. Nevertheless, recommendations to rule out mediastinal lymph node involvement differ substantially between guidelines. After a standard staging, the NCCN recommends an endoscopic (endobronchial and/or endoscopic ultrasound biopsy) or surgical staging (mediastinoscopy, mediastinotomy, and videothoracoscopy) of the lymph nodes to all patients candidates for definitive surgical resection [107]. The ESMO guidelines, however, recommend a direct surgical approach in patients without mediastinal lymph node involvement on CT scan and PET-CT scan (in these patients, mediastinal lymph node biopsy would not be required before surgery) and to perform an EBUS and/or mediastinoscopy only if there are enlarged or positive lymph nodes on CT scan and/or PET-CT scan [110].

With regard to the type of resection, lobectomy with mediastinal lymph node dissection is the recommended type of resection.

### 2.3. SBRT for early ES-SCLC

The standard treatment for patients with intrathoracic SCLC disease is concurrent CRT, while surgical resection is recommended for patients with early stage SCLC (T1-2N0). For medically inoperable patients or those who refuse surgery, definitive radiation therapy concurrently with systemic therapy is the standard approach. There is an increasing interest in evaluating the role of SBRT for inoperable early stage SCLC.

**Figure 1:**
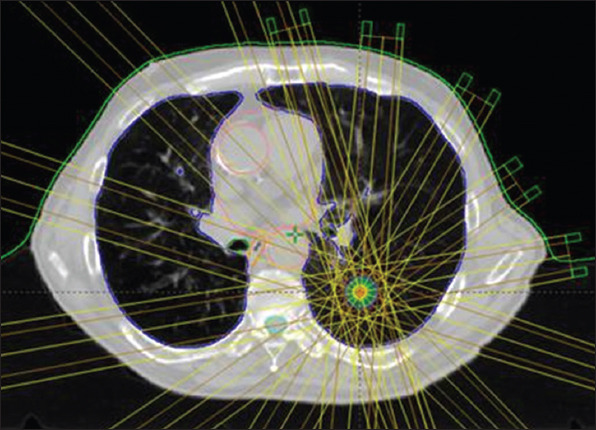
SBRT planning treatment.

SBRT, otherwise known as stereotactic ablative radiotherapy (SABR), is an advanced technique that delivers a highly conformed ablative radiation dose with great precision into a small tumor volume, usually <5 cm. It is given over a limited number of fractions, typically from 3 to 8. This technique allows minimizing the dose to surrounding normal tissues due to a very steep dose gradient, being the reason for the low toxicity (Figure 1).

SBRT has been demonstrated to be an effective treatment for medically inoperable early-stage NSCLC, with local control rates of 80-90% [111-115]. On the basis of these findings and knowing the high radiosensitivity of SCLC, SBRT has recently emerged as a potential therapeutic option in medically inoperable T1-2N0 SCLC. Principles of SBRT for early SCLC, in the absence of other data, are similar to those used for early NSCLC: Small volume (<5 cm), non-involvement of mediastinal structures, and DBE (dose biological equivalent) >100 Gy. Despite not having randomized studies, the use of this technique is increasing, while awaiting new results that validate this treatment approach.

#### 2.3.1. Review of literature’s data

Table 2 summarizes several retrospective SBRT series for patients with early stage SCLC published in recent years: Multi-institutional retrospective series, database reviews, and cases series reports.

**Table 2:** SBRT data for early stage SCLC

Study	Study type	Inclusion criteria	Cohort size	Dose	CT	PCI	Results		

							LC	OS	PFS
Videtic, 2013 [116]	RS-single center (2004-2010)	Stage I SCLC	N=6	60 Gy (3 fx) 50 Gy (5 fx) 30 Gy (1 fx)	66,6% (4/6)	66,6% (4/6)	100% (1y)	63% (1y)	75% (1y)

Ly, 2014 [115]	RS-single center (2007-2011)	Stage I SCLC	N=8	50 Gy (4 fx)	62.5% (3/8)	0%	100% (3y) 60% (3y-CT)	37% (3y)	-

		Recurrent Stage I SCLC	N=3		0%	33.3% (1/3)	100% (1y)	33% (1y)	-

Shioyama, 2015 [117]	RS-multicenter	Stage I SCLC	N=64	48 Gy (4 fx)	56.2% (36/64)	15.6% (10/64)	89.3% (2y)	76.3% (2y)	-

Stahl, 2017 [120]	RS-database NCDB (2004-2013)	Stage I SCLC	N=285	48-60Gy (3-5 fx)	45.6% (130/285)	-	35.2% (3y) 21.5% (5y)	-	-

Verma, 2017 [12]	RS-Multicenter (2005-2015)	Stage I SCLC	N=74	50 Gy (5fx)	59.2 (45/74)	23% (17/74)	96% (3y) 14.3m (no CT)	31.4m (CT) 9m (no CT)	61.3m (CT)

Shioyama, 2018 [118]	RS- database JRS-SBRTSG (2004-2012)	Stage I SCLC	N=43	36-60 Gy (3-10fx)	18.6% (8/43)	18.6% (8/43)	80.2%(2y) 47.2% (2y)* (Dm1PFS)*	72.3% (2y)	44.6% (2y)

Verma, 2019 [11]	RS-database NCDB (2004-2014)	Stage I SCLC	SBRT/CT=149 CFRT/CT=1958	45-60 Gy (3-8 fx) 45-70Gy (25-35fx)	100%	-	83.8% (1y) 29.2m (SBRT) 31.2m (CFRT)	-	-

Newman, 2019 [121]	RS-database NCDB (2004-2015)	Stage I SCLC	N (total)= 1378 N (SBRT)= 239 N (CFRT)= 1139	BED10≥100 Gy (≤8 fx)	SBRT: 35.1% (84/239) CFRT: 88.8% (1012/1139)	-	27% (5y) SBRT 26% (5y) CFRT 36% (5y) SBRT+CT 27.5% (5y) CFRT+CT	-	-

Singh, 2019 [122]	RS-RSSPR (2008-2018)	Stage I SCLC	N=21	mBED10105.6 Gy (3-5 fx)	19% (4/21)	-	100% (1y) 100% (2y) 100% (3y)	73.1% (1y) 36.6% (2y) 100% (1y-CT) 63% (1y-no CT)	85.7% (1y) 42.9% (2y)

SCLC: Small cell lung cancer; CT: Chemotherapy; PCI: Prophylactic cranial irradiation; SBRT: Stereotactic radiation therapy; CFRT: Conventionally fractionated radiation therapy; fx: Fractions; OS: Overall survival; LC: Local control; DFS: Disease free survival; Dm1 PFS: Distant metastases free survival; m: Months; y: Years; RS: Retrospective study; NCDB: National Cancer Data Base; JRSSBRTSG: Japanese Radiological Society MultiInstitutional SBRT Study Group: RSSPS: RSSearch Patient Registry; LOE: Level of evidence (From the Centre for EvidenceBased Medicine: http://www.cebm.net.)

Videtic *et al*. published in 2013 the first results of patients with ES-SCLC (T1-T2aN0) treated with SBRT with a dose of 30 to 60 Gy in 1 to 3 fractions in a small series of six patients. Four of them underwent CT and adjuvant prophylactic cranial irradiation (PCI). Local control (LC), OS and DFS obtained at 1 year were 100%, 63% and 75%, respectively. There were no regional relapses and no toxicity ≥ G3. Despite a short follow-up, the author concludes that SBRT in early stage SCLC offers excellent local control with low toxicity, being these results similar to those obtained in early non-surgical stages of NSCLC treated with SBRT [116].

The Japan Radiation Oncology Study Group (JROSG) reported in 2015 an abstract with the results of 64 patients with Stage I SCLC treated with SBRT, with a dose range of 35-60 Gy in 3 to 19 fractions. At 2 years, the LC, OS, and Progression-free survival (PFS) were 89.3%, 76.3%, and 49.3%, respectively. Eighteen patients had regional relapse and 26 had distant metastases. Female gender and the use of CT were significantly correlated with favorable survival outcome in univariate and multivariate analysis [117]. Later, the same group (JROSG) published the results of a multi-institutional retrospective series of 43 patients with Stage I SCLC treated with SBRT [118]. All patients had brain MRI/CT-scan and in 12 of them a staging ^18-FDG^PET-CT was performed. The administered dose was 30-60Gy (3-10 fractions). Eight patients received CT and PCI was performed in other eight. The 2-year OS, PFS, DmPFS (Distant metastatic Progression Free Survival), and LC were 72.3%, 44.6%, 47.2%, and 80.2%, respectively. In the univariate analysis, female sex and Stage IA were favorable prognostic factors for OS and PFS. Regional and distant metastases were 28% and 47%. This low OS percentage corresponds to a tumor with a propensity for early development of metastatic disease, but probably also related to insufficient staging and a low proportion of patients treated with CT.

In 2017, Verma *et al*. published a multi-institutional analysis of 74 patients treated with SBRT for inoperable Stage I SCLC with a median dose of 50 Gy in5 fractions, demonstrating high local control (96% at 3 years with low toxicity, 1% Grade >3). On multivariate analysis, the addition of CT was associated with improved OS and DFS. PCI was not associated with survival improvement. The relapses were most commonly distant (45.8%) and nodal (25%). After the publication of these results, clinical practice guidelines incorporated SBRT into the therapeutic strategy of Stage I SCLC for inoperable patients [119].

The National Cancer Database (NCDB) has published several analyses of patients with early-stage SCLC treated with radiation therapy: SBRT or Conventional Fraction Radiation Therapy (CFRT) with or without CT. The first of them, published the results of 285 patients with Stage T1-T2 SCLC treated with SBRT between 2000 and 2013 [120], most of them (83%) in the past 5 years. The most common dose received was 48-60 Gy in 3-5 fractions. Almost half of the patients received CT, being as neoadjuvant in 42.7% of them. Younger age and diagnosis in the initial years of the study were the only significant predictors of CT administration. After a 45.6 months follow-up, 3 and 5 years OS were 35.2% and 21.5%. OS was not affected by the time that CT was administered: Neoadjuvant or adjuvant. No data about the impact of systemic therapy in OS was analyzed in this study. In this large group of patients, we can observe the increasing use of SBRT technique despite the lack of randomized evidence.

In 2019, the NCDB analyzed 2107 T1-T2N0M0 SCLC, all of them treated with CT and RT (SBRT or CFRT). CFRT represented 92.9% and the majority was followed by CT (85%). On multivariate analyses SBRT technique was not related with an improvement of OS (31.2 m vs. 29.2 m, *P*=0.95). Predicting factors of poorer OS were advanced age, male gender, and treatment in earlier years [11].

The NCDB recently published, the results for a group of 1378 patients T1-T2N0M0 SCLC treated with radiation therapy (SBRT or CFRT). SBRT group received significantly less CT than CFRT group, 35% compared to 88%. SBRT was significantly associated with improved survival when CT was given (*P*<0.001).On multivariate analysis in the CT group, adjusting for sex, age, and tumor size, the use of SBRT trended to improve survival (*P*=0.06). SBRT was strongly associated with a survival benefit in elderly patients [121].

Singh, in 2019 identified 21 patients in the RSSearch Patient Registry (RSSPR) with medically inoperable Stage I SCLC treated with SBRT. Only four patients received CT. The 1 and 3-year local control rates were both 100%. 1 and 2-year OS were 73.1% and 36.6%, respectively. The study concluded that patient with T1N0 had better OS than T2N0, 85.7% and 33.3%, respectively. Adjuvant CT improved OS, also for Stage I SCLC over SBRT alone [122].

#### 2.3.2. Conclusions about SBRT

In light of the results obtained in these limited series (small number of patients, heterogeneous treatments, and low level of evidence), we can conclude that: As expected, SBRT offers excellent local control, more than 85%, and low toxicity, <5% Grade ≥ 3, for inoperable SCLC Stage I, with similar results when compared to NSCLC series treated with SBRT. However, OS rates are worse than those obtained in NSCLC. According to the analyzed series, the OS were 63-83%, 35-76%, and 21-26% at 1, 3, and 5 years, respectively, due to the high tendency of early spreading of SCLC; it can also be secondary to insufficient mediastinal and distant staging in many of the analyzed series, which often leads to understaging and therefore to worse results. Hence, it seems highly recommendable to systematically perform a correct regional and distant staging with an^18-FDG^ PET-CT-scan and mediastinoscopy or EBUS for all patients to correctly assess the impact of SBRT on the survival of early-stage SCLC.

2.3.2.1. Adjuvant CT

Another determining factor is the use of CT. The fact that up to 50% of recurrences are distant must be considered. In the series in which CT is analyzed, a significant increase in survival can be observed in the subgroup that receives CT, regardless of when it is given, and especially for tumors >2 cm [117,119,121]. The use of CT and its sequence (neoadjuvant/adjuvant) is heterogeneous in the analyzed series; since SBRT only requires few sessions it would not delay the beginning of CT, whereas CT would interfere with volume delimitation of SBRT. It is, therefore, preferred to perform SBRT before CT.

Tumor size at this very early stage of SCLC seems to be an important factor in all the analyzed series, as in some of them it is observed that tumors >2 cm have worse prognosis than smaller ones [119,121,122]. This should be taken into account when assessing the need for systemic therapy.

2.3.2.2. RT technique

Regarding the RT technique used, in the series in which CFRT is compared to SBRT there is a non-significant trend of a better OS in patients treated with SBRT, especially in elderly patients [11,121]. Principles of SBRT for SCLC are similar to those for NSCLC. The dose of SBRT that is used for SCLC is similar to that for NSCLC with a BED >100 Gy. There are no studies that have evaluated a different dose. Even though the series include only a low number of patients, they have reported a low toxicity (<5% G≥3) which represents a clear advantage over conventional RT, as almost all patients are elderly and have cardiorespiratory comorbidities.

2.3.2.3. PCI

Up to 50% of patients with SCLC will develop brain metastasis. Auperin’s meta-analysis [123] showed that PCI increases the rate of DFS and reduces the cumulative risk of brain metastasis by up to 25%. PCI has a Category I recommendation for localized stage SLCL. It cannot be ignored its potential neurotoxicity, therefore different approaches to reduce it has been utilized, such as the hippocampus’s protection [124,125] or the use Memantine [126].

The use of PCI in Stage I SCLC subgroup is controversial since the data that we have in retrospective surgical series show a lower risk of brain metastasis; it is estimated that between 10 and 15% will develop them [62,127,128]. Thus, expected benefit in survival could be lower. An approach that seems reasonable in the era of advanced imaging in this favorable group of patients is active brain MRI surveillance, as an alternative to PCI. The salvage whole-brain RT for treatment of brain metastases does not appear to have a detrimental effect on OS [129-131]. More studies are needed to confirm these data.

## 3. Discussion

The National Comprehensive Cancer Network (NCCN), ASTRO, ASCO, and ESMO guidelines recommend surgery as the initial treatment option in operable patients having a well-staged cT1-2N0M0 (I-IIA) of the American Joint Committee on Cancer and the International Union for Cancer Control update tumor-node-metastasis (TNM) cancer staging system. Pathologic mediastinal staging would be mandatory before surgical indication to identifying patients with a very limited disease. These recommendations are based on many small series, largely retrospective, Phase II trials, meta-analysis, systematic reviews, and propensity matched score analysis that has shown excellent survival for patients with T1-2N0SCLC who underwent surgical treatment, mainly in the setting of a multimodal approach [132]. Lobectomy with systematic lymph node dissection is the recommended type of resection.

Notwithstanding, there is a clear underuse of surgery in the treatment of ES-SCLC. Wakeam *et al*. identified 9740 patients with cT1 or cT2N0M0 SCLC cases from the National Cancer Database (2004-2013), of which only 2210 (22.7%) underwent surgery in spite of having no recognizable contraindication. Remarkably, between 2004 and 2013, the resection rates raised from 9.1% to 21.7%. The authors suggest studies addressed to found the reason for this mismatch between guidelines and practice [133]. The same group also showed that there is a large variability in the surgical indication and type of resection for ES-SCLC, but also in survival and mortality in the current clinical practice in the United States, which may represent a substantial opportunity for improvement in patients with ES-SCLC [134].

Almost 10 years have passed since Shepherd published in the Journal of Thoracic Oncology the editorial entitled “Surgery for Limited Stage Small Cell Lung Cancer. Time to Fish or Cut Bait” urging the international community to join forces to plan a prospective trial to answer the question of the role of surgery in the treatment of ES-SCLC [135].

While surgery is considered the preferred local therapy in T1-2N0 SCLC, increasing data support the role of SBRT for inoperable Stage I SCLC, transferring the favorable experience obtained on inoperable early NSCLC. SBRT shows comparable local control outcomes to surgical and CRT series, even though there are no randomized studies comparing both treatments. Use of SBRT in early-SCLC as in NSCLC seems safe and does not increase the risk of toxicity. The pattern of failure in SBRT series is regional in 25% of cases and distant in 50%. To correctly assess the impact of SBRT on the survival of stage I SCLC is highly recommendable to systematically perform a correct regional and distant staging: ^18-FDG^ PET-CT scan, mediastinoscopy, and/or EBUS.

SCLC is a tumor with a high trend to early spreading and it is unknown why a high percentage of patients did not receive chemotherapy. The data from surgical, CRT, and SBRT series support multimodal treatment with neoadjuvant or adjuvant chemotherapy and PCI even in early-SCLC. Systemic treatment showed significant increase in OS in both surgical and SBRT series. According to the analyzed data, the range of benefit in OS at 5 years goes from 10% to 30%, with a significant advantage in the chemotherapy group. The sequence of systemic treatment – neoadjuvant/adjuvant – showed no significant impact in OS. However, in the context of a local primary treatment, surgery or SBRT, it seems more reasonable that it be adjuvant.

PCI is associated with a significant reduction of brain metastases in SCLC. The selected group of early SCLC has a lower risk of brain metastasis; which is estimated between 10 and 15%. The impact on survival is lower than in other stages. The most appropriate approach is unclear. Active brain MRI surveillance may be an alternative option to PCI, especially in very early stage (T1N0M0) SCLC. More studies are needed to confirm these data.

According to the current data, it is difficult to compare surgery and SBRT treatments. The patients in each group of treatment have different characteristics in terms of age and comorbidities. Surgery started earlier than SBRT as a local treatment of early SCLC. Surgical series are extensive, with many patients analyzed, long-term follow-up, and robust conclusions; on the other hands, SBRT is a new emergent treatment for early stage SCLC; therefore, current available studies are retrospective, heterogeneous, and with a small number of patients. Despite this, preliminary results of SBRT are promising and warrant future research. The advantage of SBRT over surgery is that it can be performed in elderly patients with altered respiratory function or other comorbidities, without increasing toxicity. Local control is similar for both treatments, although regional relapse was superior in SBRT series, probably due to a mediastinal understaging in SBRT series. Therefore, it is difficult to compare both treatments and should be further investigated in randomized clinical trials.

**Figure 2:**
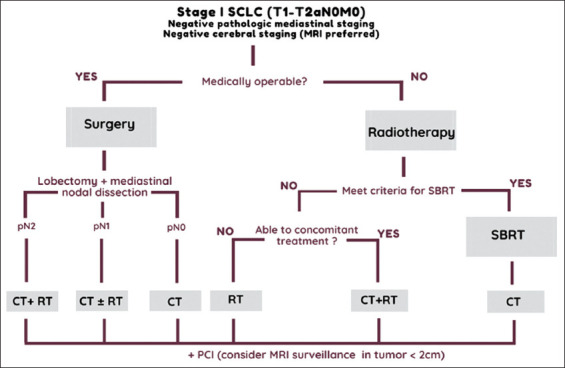
Proposed algorithm for treatment of ES-SCLC. SBRT: Stereotactic body radiotherapy; CT: Chemotherapy; RT: Conventional radiotherapy (concomitant or sequential); PCI: Prophylactic cranial irradiation; MRI, Magnetic resonance imaging.

Taking into account the data analyzed, the limitations of the series reviewed, and the recommendations of the international guidelines, we propose our approach in the management of T1-2N0 SCLC considering: Staging, operability, criteria for SBRT, ability to systemic therapy and benefit of PCI (Figure 2).

## 4. Conclusion

T1-2N0M0SCLC represents a highly selected group of patients who, despite having a very localized disease, requires a multimodal approach due to the aggressive nature of the disease. In spite of the absence of randomized studies, the current data and guidelines recommend surgical treatment of well-staged early SCLC (cT1-2N0M0) in patients fit enough for surgery. Lobectomy plus systematic nodal dissection is the preferred type of resection. Nowadays, RT is the therapeutic option in those with surgical contraindication, despite the lack of high quality clinical evidence; SBRT is an emerging technique that is safe and effective for inoperable early stage SCLC. All patients should receive systemic therapy due to the tendency of SCLC to metastasize early, with the aim of increasing OS. PCI must be evaluated individually by a multidisciplinary team.

### Conflict of Interest

The authors declare that they have no conflict of interest.
